# Anxious and depressive symptoms mediate the influence of sleep quality on suicidality in young adults

**DOI:** 10.3389/fpubh.2024.1322069

**Published:** 2024-01-11

**Authors:** Corinna Slanitz, Jürgen Fuchshuber, Andreas Fink, Human-Friedrich Unterrainer

**Affiliations:** ^1^Department of Psychiatry and Psychotherapeutic Medicine, Medical University Graz, Graz, Austria; ^2^Institute of Psychology, University of Graz, Graz, Austria; ^3^Department of Psychoanalysis and Psychotherapy, Medical University Vienna, Vienna, Austria; ^4^Center for Integrative Addiction Research (CIAR), Grüner Kreis Society, Vienna, Austria; ^5^Institute of Religious Studies, University of Vienna, Vienna, Austria; ^6^Faculty of Psychotherapy Science, Sigmund Freud University, Vienna, Austria

**Keywords:** anxiety, depression, sleep quality, somatization, suicidality

## Abstract

This study investigated how sleep quality affects the global severity of psychiatric symptoms, including suicidality, in young adults. Poor sleep quality has a significant impact on mental health and should therefore be given special attention in suicidal treatment. 1,214 participants (914 females; age: *M* = 25.81, *SD* = 6.35) completed the Brief Symptom Inventory (BSI-18), the Scale for Suicidal Experience and Behavior (SSEV), and the Pittsburgh Sleep Quality Index (PSQI) via an online survey. Correlation statistics and path analysis were conducted for data evaluation. Thereby, anxiety and depression but not somatization mediated the relationship between sleep quality and suicidality. Our findings confirm the putative link between diminished sleep quality and increased suicidality and may lead to an early detection of suicidal behavior.

## 1 Introduction

Suicide is an important public health issue worldwide and one of the leading causes of loss of life (1). Suicidality is a complex process that involves a series of pathways and mechanisms from initiation of suicidal ideation, to planning, and finally, to attempting suicide (2). Death by suicide and suicidal ideation and behavior among young adults are major public health concerns. A recent United States-study among young adults has shown national trends of suicidal behavior and the results indicate a growing number of young people with suicidal thoughts, suicidal ideations, and behavior (3). Well-established risk factors for suicidal ideations and behavior are the presence of mental disorders, especially mood disorders, like anxiety and depression (4, 5), somatization (6), and sleep quality (7). There is evidence to suggest that individuals who are at risk for suicide often experience sleep problems. Bad sleep quality and other sleep disturbances are common among people with mental health issues, and addressing these problems may contribute to overall suicide prevention efforts. Nevertheless, existing literature provides limited information on the correlation between suicidality and poor sleep quality and few studies have examined sleep quality in relation to suicidal behavior. Consequently, this study places special emphasis on identifying and examining possible causes that are responsible for the development of suicidality, with a special focus on bad sleep quality in young adults. Specific research can help to identify specific sleep-related risk factors associated with an increased risk of suicidal thoughts and behaviors (8).

Many studies suggests that good sleep is important for maintaining general health and wellbeing and poor sleep quality has been shown to be associated with bad health outcomes (9). Symptoms of bad sleep quality are an integral component of human life and can impact several variables of human physiology and psychology, and some studies assume that bad sleep quality can even be associated with suicidal thoughts and behaviors (10, 11).

One factor found to be associated with increased vulnerability for suicidal ideation or suicidal behavior is bad sleep quality in terms of sleep disturbances (12). Other specific sleep parameters are also associated with increased likelihood of suicidal thoughts, attempts, and death by suicide, including short self-reported sleep duration and poor sleep quality (13).

Furthermore, bad sleep quality is also a symptom among other affective disorders, like depression and anxiety (14). Moreover, poor sleep quality and somatization often have a reciprocal relationship, with each influencing and exacerbating the other. Persistent somatic symptoms can contribute to sleep disturbances, while insufficient or disrupted sleep can intensify somatic complaints (6). Somatization is defined as the externalization of psychological distress through the presentation of physical symptoms. This phenomenon frequently coexists with diverse mental health disorders, encompassing depression and anxiety disorders (15). More recently, poor sleep quality was associated with higher risk of depression in both sexes, and improvement in sleep quality was associated with a reduction in depressive symptoms. It has also been observed that suffering from depressive symptoms is significantly related to decreased sleep quality (11, 14).

Mental disorders such as depression or anxiety have been identified as one of the major public health problems in general, but also in suicide behavior (16). Post-mortem studies which compared 302 consecutive individuals, who made medically serious suicide attempts, to more than 1,000 random controls, have a probability of 33.4% for individuals with mood disorders (16). According to ICD-10, depressive disorders can be characterized by decreased drive, reduced activity, depressed mood, loss of interest, exhaustion, guilt, bad sleep and even suicidal thoughts, intentions, and behavior (17). Corresponding to ICD-10, common symptoms of anxiety disorders include excessive worry, restlessness, fatigue, muscle tension, and sleep disturbances (17). Especially symptoms of depression and bad sleep quality in young adults are common, affect each other, and are highly comorbid (16). Moreover, most symptoms of anxiety disorders were significantly associated with bad sleep quality scores (18). A strong positive association between depressive and anxiety disorders, sleep quality, and suicidality may be considered generally confirmed (19).

Somatization disorders are characterized by the presence of multiple, recurrent, and frequently changing physical complaints that have no apparent medical cause (17). Symptoms of somatization include pain, gastrointestinal problems, neurological complaints, and sleep problems (15). Physical discomfort and worrying about physical symptoms can contribute to sleep disturbances, leading to difficulties in falling asleep or staying asleep (16). The perception of physical symptoms, absent clear physiological etiology, can be influenced by negative thought patterns and cognitive biases. The conversion of psychological distress into physical manifestations serves as a non-verbal modality for expressing deep emotional suffering, thereby being correlated with heightened vulnerability to suicidality (15).

The necessity of this study is to improve the understanding of factors and mechanisms underpinning pathways to suicidal thoughts and attempts in young adults. Unlike many other risk factors, sleep disorders are generally readily treatable, and may can help to develop effective suicide-focused interventions and to intervene before an attempt. As an early alarm signal, sleep disorders may be particularly useful for research and may be an important factor in suicide interventions. In line with this, the purpose of this study is to further investigate the association between decreased sleep quality and suicidal intentions in young adults and furthermore, may identify sleep disturbances as a risk factor in suicidality.

## 2 Methods

### 2.1 Participants and procedure

A total sample of 1,214 young adults (age range = 18–40 years) from a non-clinical population took part in the survey over a period of 8 weeks. The study was conducted online via social media channels and in co-operation with psychologic institutions. Good German language skills were an inclusion criterion and written consent was obtained from all participants. The initial sample comprised a total of 1,436 participants and 222 participants were excluded due to incomplete questionnaires. A total of 1,214 people were analyzed, 914 (75.3%) were female, and 300 (24.7%) were male. The average age was *M* = 25.8 years (*SD* = 6.35). The overall sleep quality score was *M* = 8.11 (*SD* = 3.69). BSI-18 scores included depression (*M* = 1.20, *SD* = 1.00), anxiety (*M* = 0.91, *SD* = 0.80), and somatization (*M* = 0.65, *SD* = 0.59) and 6 (0.5%) participants reported a suicide attempt in the past 4 weeks.

### 2.2 Research tools

The German version of *Brief Symptom Inventory* (*BSI-18*) (20) was used to assess psychological distress and psychiatric conditions over the past 7 days. The inventory includes the subscales of depression, anxiety, and somatization. The items of the BSI-18 can be answered on a five-point Likert scale (from 0 = strongly disagree to 4 = strongly agree). By summing all three scale scores, a Global Severity Index (GSI) can be formed, which provides information about the overall severity of general psychiatric symptoms. The BSI-18 always showed satisfactory internal consistency with Cronbach’s α ranging from 0.70 to 0.89 and 0.93 for the total score, respectively.

The *Scale for Suicidal Experience and Behavior* (*SSEV*) (21) was used to measure the frequency and intensity of passive and active suicidal ideation as well as suicidal intentions, suicidal impulses, suicidal plans, and previous suicide attempts. The SSEV is a self-report instrument that requires items to be answered on a six-point Likert scale (from 0 = never to 5 = every day) and contains nine items. Several studies have found a satisfactory Cronbach’s *α* between 0.73 and 0.89.

The *Pittsburgh Sleep Quality Index* (*PSQI*) (22) was used for the assessment of sleep problems. The PSQI is a self-rating questionnaire with 19 questions and seven component scores: sleep quality, sleep latency, sleep duration, habitual sleep efficiency, sleep disturbances, use of sleeping medication, and daytime dysfunction. Each component score is rated from 0 to 3 (0 = not in the past month to 3 = three or more times per week). The global score ranges from 0 to 21, with higher scores indicating poorer sleep quality. Cronbach’s α ranging from 0.77 to 0.86 in different studies. The cut-off value of PSQI is ≤5, higher scores indicate disturbed sleep quality.

### 2.3 Statistical analyses

SPSS 27.0 was used for data management, descriptive statistics, and bivariate correlations. Path and mediator analysis were conducted with AMOS 28. To investigate the relationship between sleep quality with depression, anxiety, and suicidality, Pearson’s correlations were calculated. As the requirement of normal distribution for the use of parametric statistical methods was violated for some variables, equivalent non-parametric procedures were conducted in these cases. Bonferroni corrections were applied for all analyses to prevent type-I error inflation due to multiple testing. Statistical significance was set at *p* < 0.05. In order to control for α-inflation, the level of significance was set to *p* < 0.01 in ANOVAs and Pearson’s correlations, while values of *p* < 0.05 were marked as tendencies, but where not further interpreted. The normal distribution of the residuals was also checked using a histogram and normal distribution plot and were also considered to be given. Regarding path analysis, a pruning strategy was applied in which non-significant paths of an initial model—which estimated all possible associations between variables—were removed. The model was controlled for age and gender effects. Goodness-of-fit was assessed with a maximum likelihood estimation in AMOS. To test for mediation and indirect effects, a bootstrap was performed with a bias-corrected confidence interval of 95% and 1,000 bootstrap samples (23). Due to severe non-normality of the investigated scales, logarithmic transformation was implemented. In accordance with Kline (24), the following fit-indices were considered as markers for an acceptable model fit: (a) The comparative fit index (CFI) > 0.90; (b) Tucker-Lewis index (TLI) relative fit index >0.90; (c) the square root error of approximation (RMSEA) < 0.08 and the upper bound of its 90% confidence interval < 1.

## 3 Results

### 3.1 Demographics and sample characteristics

As described before, a total of 1,214 subjects were studied. 914 (75.3%) were female, 300 (24.7%) were male. The mean age was *M* = 25.8 years (*SD* = 6.35). Regarding to education level, 615 (50.7%) reported high school graduation, 388 (31.9%) had a university degree (Bachelor, Master, PhD), 160 (13.2%) had an apprenticeship, and 51 (4.2%) had completed compulsory education. 475 (39.1%) of the individuals were currently in full-time study, 298 (24.5%) were studying alongside part-time employment, 266 were employed full-time, 129 were employed part-time, and 46 (3.8%) were unemployed. 145 (11.9%) were taking psychotropic drugs in the past 4 weeks and 6 (0.5%) individuals reported a suicide attempt in the past 4 weeks. 146 (12%) had a pre-existing condition such as metabolic, muscular, or cardiovascular disease.

### 3.2 The relationship between sleep quality and psychiatric symptoms

The overall sleep quality score was assessed with the PSQI (*M* = 8.1, *SD* = 3.69). In comparison to the norm sample of 1,049 participants between 15- and 50-year poor sleepers had values between 6 and 10 points. Mean values of BSI-18 and GSI in this study were compared to the norm sample of BSI-18. Depression (*M* = 1.20, *SD* = 1.00), anxiety (*M* = 0.91, SD = 0.80), and somatization (*M* = 0.65, SD = 0.59) were significantly lower (*p* < 0.001) than in the norm sample [depression (*M* = 2.37, SD = 3.43); anxiety (*M* = 2.47, *SD* = 2.57); and somatization (*M* = 1.50, *SD* = 2.16)]. Correspondingly, the GSI was significant lower (M = 2.75, SD = 2.06) than in the comparable norm sample (*M* = 6.32, *SD* = 6.90, *p* < 0.001). In this study, SSEV values were quite low and only 0.5% participants reported a suicide attempt in the past 4 weeks. Higher values in total score for SSEV indicate increased suicidal experience. There are no norm or cut-off values. Pearson correlations were calculated to examine the interactions with GSI and subscales depression, anxiety, and somatization as well as suicidality and sleep quality.

As demonstrated in Table 1, GSI was strongly positively associated with depression (*r* = 0.89; *p* < 0.001), anxiety (*r* = 0.88; *p* < 0.001), somatization (*r* = 0.78; *p* < 0.001), and sleep quality (*r* = 0.54; *p* < 0.001). Furthermore, GSI was observed to be slightly higher in females (*r* = −0.14; *p* < 0.001) and correlated mildly negatively with age (*r* = −0.21; *p* < 0.001). Suicidality was strongly correlated with GSI (*r* = 0.55; *p* < 0.001) and among the sub-scales, especially strongly correlated with depression (*r* = 0.59; *p* < 0.001), followed by anxiety (*r* = 0.41; *p* < 0.001), somatization (*r* = 0.37; *p* < 0.001), and diminished sleep quality (*r* = 0.29; *p* < 0.001). Correspondingly, sleep disorders showed moderate correlations with depression (*r* = 0.48), anxiety (*r* = 0.47), and somatization (*r* = 0.44; all *p* < 0.001).

**Table 1:** Intercorrelations among examined variables.

Variable	1	2	3	4	5	6	7	8
1. GSI	-							
2. Suicidality	0.55^**^	-						
3. Depression	0.89^**^	0.59^**^	-					
4. Anxiety	0.88^**^	0.41^**^	−0.65^**^	-				
5. Somatization	0.78^**^	0.37^**^	0.53^**^	0.61^**^	-			
6. Sleep Quality	0.54^**^	0.29^**^	0.48^**^	0.47^**^	0.44^**^	-		
7. Gender	−0.14^**^	0.04	−0.09^*a^	−0.17^*a^	−0.11^*a^	−0.15	-	
8. Age	−0.21^**^	−0.15^**^	−0.21^**^	−0.16^**^	−0.17^**^	0.01	−0.07^*a^	-

*N* = 1,214; Gender was coded as 0 = female and 1 = male; ^*^p < 0.05, and ^**^p < 0.001; ^a^non-significant after Bonferroni correction; GSI, Global severity index.

### 3.3 The influence of sleep quality on psychiatric symptoms and suicidality

**Figure 1:**
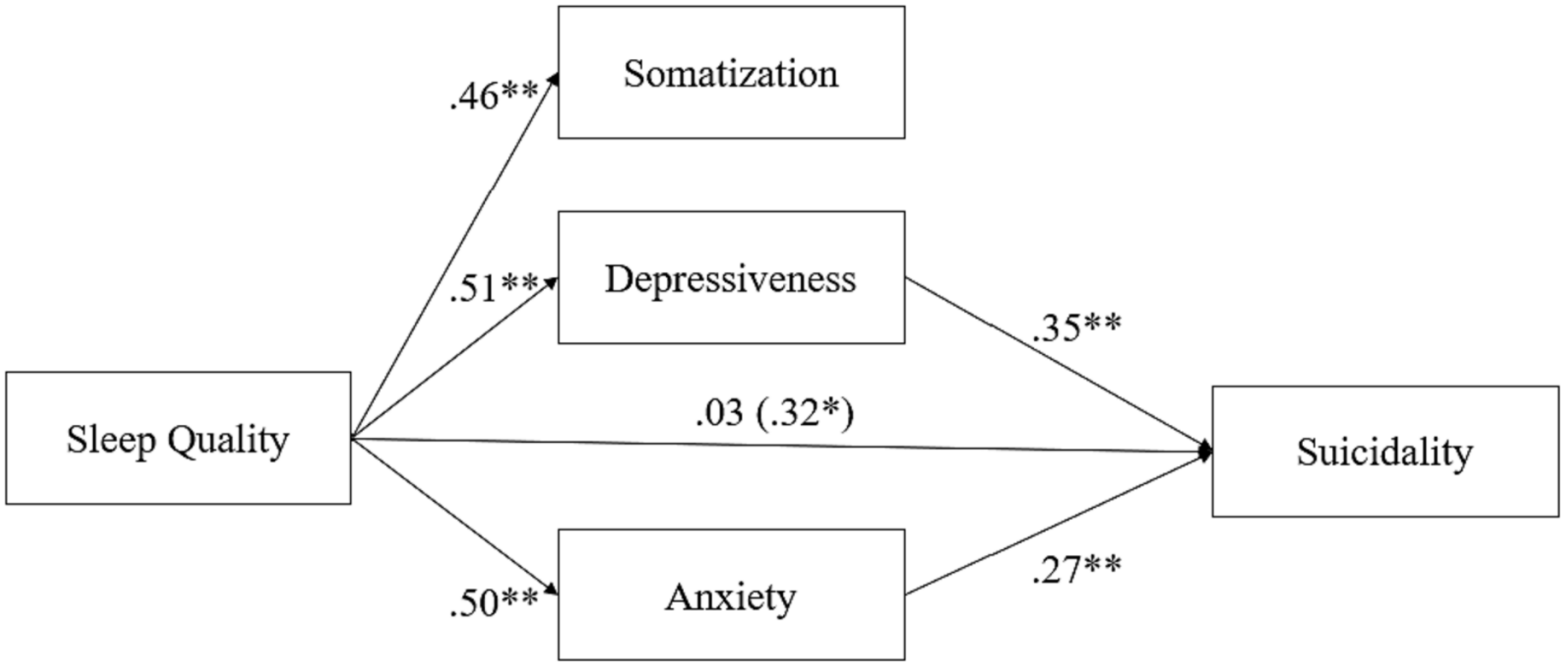
Mediation effect of psychiatric symptoms (So, De, and An) on the relationship between sleep quality and suicidality.

An initial pruning step trimmed the non-significant association between somatization and suicidality (*p* > 0.05). This resulted in the model displayed in Figure 1. The model indicated an acceptable global fit with the following indices: RMSEA = 0.07 (90% CI, 0.05; 0.09); TLI = 0.95; CFI = 0.98.

As shown in Figure 1, sleep quality was significantly associated with somatization (*β* = 0.46; *p* < 0.001), depression (*β* = 0.51; *p* < 0.001), and anxiety (*β* = 0.50; *p* < 0.001), while its association with suicidality was non-significant (*β* = 0.03; *p* > 0.05). Depression (*β* = 0.35) and anxiety were significantly associated with suicidality (*β* = 0.27; both *p* < 0.001). Further inspection revealed a significant indirect effect of sleep quality on suicidality (*β* = 0.32; *p* < 0.01) fully mediated via depression and anxiety.

## 4 Discussion

This study aimed to further contribute to the ongoing investigation of the relationship between psychiatric symptoms and decreased sleep quality in young adults, an age group in which sleep problems have been shown to be particularly problematic (25). Consistent with our hypotheses, sleep disturbances were observed to be associated with an increased number of psychiatric symptoms, including suicidality. Moreover, this study also underlined the very broadly confirmed association between suicidality and anxious and depressive symptoms. This finding is consistent with the fact that most suicide attempts are committed by individuals suffering from major depression (26) and that suicidal ideation increases with the depressive spectrum (27).

In addition, suicidal thoughts and attempts are frequently described as a symptom of depression, as evidenced by the significant correlation between depression and suicidality (*p* < 0.001). Accordingly, there is ample evidence that sleep disturbance is a hallmark of major depressive disorder and that decreased sleep quality is a risk factor for the development of depression (28). In our study, we observed a significant mediation effect of anxious and depressed symptoms on the relationship between sleep problems and suicidality, which is also consistent with the literature (11). No mediation effect was found here for somatization, which needs further investigation.

### 4.1 Limitations and future research perspectives

The causes of suicidality are a complex interaction of many factors. As a caveat, suicidal ideation was quite low in this study, and only 0.5% had attempted suicide in the previous 4 weeks. Perhaps this is one reason why the other variables studied did not have a large effect on suicidality and had low correlations with SSEV. In conclusion, the level of psychiatric symptom distress was significantly associated with decreased sleep quality. However, in this study, the overall sleep quality score was quite bad. Although depression, anxiety, and somatization scores were moderate in this study, our findings point to the fact, that sleep quality may have a greater impact on mental health than expected. Further research, particularly in clinical samples, is now needed to further characterize the role of sleep quality in mental health problems.

Moreover, the small inclusion criteria may limit the generalizability of results to a broader population. This can lead to challenges in applying findings to diverse patient groups and may overlook potential variations in treatment responses among different subpopulations. Further research, particularly in clinical samples, is now needed to further characterize the role of sleep quality in mental health problems and suicidality.

Significantly more women than men participated in our study, which naturally makes it difficult to generalize the results. In line with the recent literature (29), it can be assumed that women suffer from sleep problems to a greater extent than men, so that the prevalence of sleep problems in the general population could be overestimated. Further analyses of the influence of gender on sleep quality would be important in any case.

## 5 Conclusion

The main aim of the study was to offer a valuable insight between the relationship of suicidality and bad sleep quality in young adults. The study shows the importance of identifying young adults with poor sleep quality as a potential risk factor for suicide and could enhance risk assessment in clinical settings. Moreover, developing targeted interventions to improve sleep quality might be crucial in preventing or reducing suicide risk.

## Data availability statement

The raw data supporting the conclusions of this article will be made available by the authors, without undue reservation.

## Ethics statement

The studies involving humans were approved by Ethics committee of the University of Graz. The studies were conducted in accordance with the local legislation and institutional requirements. The participants provided their written informed consent to participate in this study.

## Author contributions

CS: Writing – original draft. JF: Writing – review & editing. AF: Writing – review & editing. H-FU: Writing – review & editing.
